# Enabling decision-making and innovation in learning health systems through simulation modelling

**DOI:** 10.1177/08404704251348857

**Published:** 2025-06-12

**Authors:** Lysanne Lessard, Antoine Sauré

**Affiliations:** 1 56004 University of Ottawa, Ottawa, Ontario, Canada.; 2 Institut du Savoir Montfort – Research, Ottawa, Ontario, Canada.

## Abstract

Canadian healthcare systems require profound transformations to enhance patient experience, improve population health, reduce costs, and improve the work life of healthcare providers. Learning Health Systems (LHSs) are an approach for undertaking this transformation in an effective, efficient, and sustainable manner with digital technologies as a key enabler for change. However, the successful implementation of a LHS brings with it challenging and potentially risky changes to clinical practices and operations. Simulation modelling is an advanced analytics technique particularly well-suited for informing decision-making and planning prior to and during the transformation of complex systems such as LHSs. Yet, despite the use and demonstrated benefits of simulation modelling in many different industries including healthcare, its application in the context of LHSs has received limited attention. In this article, we discuss how simulation modelling can be leveraged to support better-informed, lower-risk decisions and innovation in LHSs.

## Introduction

A Learning Health System (LHS) is a vision for transforming current health systems and healthcare provider organizations into high-performance entities able to provide effective and safe care at lower costs while providing excellent patient and care provider experience.^1,2^ This vision is driven by the availability of large health-related datasets, such as those contained in electronic health records, combined with the possibilities offered by major technological advances in digital infrastructures and data processing capabilities to support timely and evidence-based decision-making.^3^ Digital technologies and data can thus be leveraged to support rapid learning cycles moving from raw data to knowledge creation and changes in practice, the results of which can then be used to initiate new learning cycles in an ongoing improvement approach.^4^

In Canada, LHSs have been identified as a key strategy for modernizing our health systems and addressing long-standing issues including unnecessary delays in access to care, siloed care delivery, human and financial resource constraints, and slow uptake and scaling-up of innovations.^1,5^ Examples of LHS initiatives in Canada include the Alliance for Healthier Communities, a community-governed primary healthcare organization in Ontario that has reorganized itself as a LHS^6^ and ACCESS Open Minds, a LHS aiming to improve youth mental health services across Canada through a pan-Canadian online data repository that provides help to youth and their families.^7^ LHSs can also be found at the regional and organizational level, such as a LHS initiative driven by the Ottawa Hospital that aims to redesign lung cancer care processes in Eastern Ontario.^8^ The LHS model is also being proposed as a means to address pressing issues related to core Canadian health systems values such as improving Indigenous health,^9^ eliminating health inequities,^10^ and advancing environmental sustainability.^11^

Nevertheless, transforming a health system into a LHS is challenging due to the inherent complexity of healthcare at scale. Successfully transforming a system into a LHS requires redesigning the whole system or organization around learning processes rather than instigating unconnected initiatives or projects.^12^ This entails managing several learning cycles, each focusing on a specific clinical or operational problem, each moving at its own speed.^13^ Understanding the causes and consequences related to a diverse set of performance metrics and clinical outcomes within these learning cycles is key to their success. But since complexity tends to create opacity, changes to clinical and operational practices that are inherent to a LHS could increase the risk of unintended outcomes.

Implementing a shared infrastructure of digital, physical and human resources, methods, incentives, ethics, culture, governance, and more is a key enabler for successfully managing such interconnected yet distinct learning cycles.^13^ Making data available on all aspects of care and operations is also required to support clinical and operational decision-making in such an infrastructure.^13^ However, data availability is only the first step in creating a holistic view of a LHS and its learning cycles. Methods providing the ability to understand how a complex system works and what causes inefficiencies, errors, and lack of performance are needed to prevent and mitigate the risks that could arise from ongoing changes inherent to a LHS.

A useful approach for supporting transformation efforts and ongoing LHS processes is simulation modelling, which is a family of methods that rely on software tools to represent a system at different scales. These representations enable the identification of causes of observed events or problems and provide insights into the potential outcomes of anticipated changes. Simulation modelling is particularly well-suited for representing and generating insights for complex systems by facilitating the analysis of alternative downstream decisions on variables such as costs or outcomes.^14^ The analyses conducted using simulation models can thus inform decision-making and planning prior to making real-life changes to existing digital infrastructures, governance structures, the allocation of human and financial resource, clinical practices, and more.

## Simulation modelling in healthcare

Simulation modelling involves creating a computer representation (i.e., a model) of a real process within a system in such a way that it captures its essential properties and relationships. Such a model provides an analysis tool to explore how a system is functioning, evaluate the impact of different potential interventions, and determine how the impact may be affected by other factors or features within the system itself.^15^ While descriptive and predictive analytics methods, respectively, focus on *what happened* and *what will happen*, simulation modelling is part of prescriptive analytics and helps to answer questions related to *what may happen* and *why it happened*. Moreover, simulation modelling offers more comprehensive support to decision-making than predictive techniques such as Machine Learning by providing the ability to evaluate alternative system configurations that differ in internal and external aspects.

Simulation modelling (hereafter “Simulation”) was first used in the defence industry in the 1950s, where early models were built using programming languages as FORTRAN and run in mainframes.^16^ Since then, simulation has evolved and been applied to modelling and improving processes in many different industries. In healthcare, applications of simulation have involved a wide range of operational and clinical decision-making problems^17,18^ (see Table 1).

**Table 1.** Overview of simulation methods.

Method	Description	Use	Examples of support to decision-making
MCS	Models the possible outcomes of an uncertain event through repeated random sampling	Evaluate functions (e.g., performance metrics) that are impossible to evaluate through direct analytical methods^20^	Patient-treatment matching^21^; cancer screening and treatment^22^; mental health status monitoring^23^; setting maximum recommended wait time targets^24^; diagnostic imaging operations^25^; operating room scheduling^26^; capacity planning^27^
ABS	Models the behaviour of individuals (agents) and their interactions within a system or population	Understand how small changes in behaviour and interaction influence the overall system or population’s dynamics and emergent behaviours^28^	Mental health treatment coverage and provision^29^; targeting public health interventions^30^; optimal treatment selection^21^
DES	Models the operation of a system, where entities flow through a number of activities, over a chronological sequence of events	Determine the impact of resource availability on waiting times and waitlists^28^	Blood supply chain management^31^; design and operational management of mass vaccination centres^32^; chronic obstructive pulmonary disease management^33^; stroke treatment delivery^34^; diagnostic imaging operations^35^; capacity planning^36^; capacity planning and appointment scheduling^37^; cancer screening planning^38^; preventing HIV transmission^39^; treatment of renal failure^40^; screening for diabetic retinopathy^41^
SD	Models the behaviour of a complex, dynamic system over time in terms of stocks (e.g., resources and people), flows between stocks and information that determines the values of the flows	Obtain long-term, aggregate-level insights about the response of the system to strategic decisions or decisions affecting a whole population^28^	Hospital capacity planning^42^; mental health intake and treatment management^29^; management of AIDS epidemic^43^; COVID-19 response^44^; chlamydia screening^45^

Four common simulation techniques used to improve healthcare systems are Monte Carlo Simulation (MCS), Agent-Based Simulation (ABS), Discrete-Event Simulation (DES), and System Dynamics (SD).^19^ These techniques are described in Table 1, which also provides examples of their application.

Simulation offers significant advantages for analyzing and understanding the behaviour of complex systems. Indeed, it is much easier, faster, more cost-effective, and safer to assess the potential outcomes that changes in a process may introduce than to directly intervene in a real system. Doing so encourages a “let’s try” attitude that fosters insights and innovation and supports better-informed, lower-risk decisions. Simulation models are also very helpful in understanding the broader impact of changes by highlighting system interdependencies and emerging behaviours, enabling holistic thinking. Related applications include examining the viability of new processes, conducting cost-benefit analysis, exploring system redesign options, examining key performance metrics in both the current and future states of the system, assessing implementation plans and risk, and disseminating the working of newly redesigned processes.^16^ Moreover, the mere process of building a simulation model is often beneficial, providing stakeholders with insights about causal determinants of a given problem, required trade-offs of a potential solution, and relevance of chosen metrics to measure outcomes.^46^

Positive results associated with the use of simulation include reduced waiting times, earlier appointment confirmation times, higher appointment reliability, increased patient and care provider satisfaction, improved resource utilization and coordination, reduced overtime, balanced staff workload, decreased clerical rework, reduced costs, increased service levels, and increased adherence to clinical guidelines for diagnosis and treatment. However, despite the successful use of simulation techniques in healthcare and their proven ability to support understanding and decision-making at a system level, their uptake in the context of LHSs is still in early stages. The rapid emergence of LHSs across Canada is providing a timely opportunity to introduce simulation alongside other advanced analytics methods and digital technologies to support the development and implementation of LHSs.^4^

## Supporting clinical and operational decision-making in LHSs

A core characteristic of a LHS is its organization around learning cycles aiming to address issues that impede the achievement of the Quadruple Aim by speeding up the implementation of innovations and ensuring their continued improvement.^2^ Learning cycles within a LHS are composed of three core processes: Data to Knowledge (D2K), where new knowledge is created through accessing and processing clinical and operational data; Knowledge to Performance (K2P), where new knowledge and insights are used to change processes and practices to achieve desired outcomes; and Performance to Data (P2D), where changes to processes and practices are evaluated.^13^ Learning cycles are thus inherently iterative as the P2D process ensures that changes in performance are documented and analyzed to identify next steps for improving a clinical or operational problem of interest.

This view of LHSs helps to understand how simulation can support LHS leaders in making effective decisions by pinpointing its role in specific learning processes. In the D2K learning process, simulation modelling can support decision-making by helping to understand a problem and its causes.^47^ Examples of this application of simulation techniques in the context of LHSs include the use of ABS to model a collaborative LHS as a population of patient- and doctor-agents with the goal of improving the patient-treatment matching process and its implementation while considering engagement, information and knowledge sharing among agents.^21^ More recently, MCS was used to generate synthetic patients for building an artificial data-centric, ML-enabled risk prediction LHS using lung cancer and stroke as examples.^48^ These examples show the ability of simulation to create new understandings from data, whether at the individual, population, or system level.

Simulation can also be at the core of implementation strategies part of the K2P learning process by providing decision-makers with the ability to examine underlying assumptions, consider alternative strategies, and anticipate their consequences,^46^ hence helping to select the best option for addressing a problem.^47^ For example, simulation can be used to identify, predict, and prevent errors prior to implementing medical guidelines.^49^ MCS (specifically, a Markov model) was used to conceptualize a patient journey for seniors receiving care in their homes in order to evaluate potential interventions and determine which interventions should be subsequently piloted as part of the following stages of a LHS cycle.^50^ Simulation can also be used to identify conditions for successful implementation, for example, when designing coordinated service infrastructures (e.g., integrated practice units) in the context of HIV-AIDS care.^51^ Essentially, in cases where the implementation of innovations and improvements may have unforeseen effects, simulation offers a valuable analysis tool for anticipating and preventing negative impacts on efficiency, quality, and outcomes.^49^

Simulation techniques may also help to plan P2D learning processes. For example, MCS (specifically, semi-Markov models) have been used to model populations at both the aggregate and individual levels to perform survival and cost-benefit analyses of varied decision policies.^52^ In another study, researchers performed a DES analysis of a stroke guideline that had already been implemented, showing that its cost-benefit was marginal while a much greater cost-benefit could have been achieved by reducing patients’ time from stroke onset to hospital arrival by 30 minutes.^53^ Using simulation prior to implementing the guideline could have saved costs while increasing the number of treatable patients, thus directing resources to a more promising performance target. These examples show that using simulation within the P2D learning process can answer the call for methods that enable the assessment of a LHS’s performance and the conditions in which it may best operate.^54^

Beyond its use in facilitating specific learning processes, simulation can support decision-making that relates to a LHS’s infrastructure or issues that span multiple learning processes. This includes clinical decision-support models designed to assist individual clinicians and their patients with decisions regarding their care. For example, clinical decision-support models developed using simulation methods can be used to plan for or replicate clinical trials, helping to estimate clinical performance metrics such as morbidity and mortality rates and cost.^52^ Simulation models are known for their usefulness in assessing the impact of introducing a medical, operational, or technological innovation on system-wide behaviour and performance.^14^ Policy decision-support models can thus be developed to more broadly evaluate whether particular healthcare technologies or other innovations should be implemented within the context of an organized healthcare system such as a LHS.^14^ More generally, simulation models can support operational decision-making by evaluating the behaviour of a health system using measures such as flow time, wait time, throughput, and resource utilization.^52^

## Innovating through simulation

Innovation should be a core value to foster rapid learning, but in practice, innovation is difficult to incorporate and evaluate in a LHS.^54^ Embedding simulation in novel digital technologies could increase their value and capabilities, facilitating their uptake.

Two transformative digital technologies that are being embraced in healthcare are process mining and digital twins.^55,56^ The first is a data-driven methodology used to analyze event logs from information systems to gain insights into how real processes operate, identify inefficiencies, and optimize workflows.^57^ By offering data-driven insights into how healthcare processes are actually executed, process mining can be used to create rich data and representative process models for simulation. Simulation models can then be used to inform process optimization efforts in a manner that increases system-wide performance.

Digital twins are virtual replicas of physical systems that dynamically reflect their real-world counterparts.^58^ While both simulation and digital twins involve replicating real-world systems in a virtual environment, a digital twin goes beyond simulation by connecting a virtual model to its physical counterpart in real-time, enabling continuous data exchange and two-way communication. Embedding simulation models in this environment not only allows to rigorously test and evaluate the potential impact of changes to the physical system before their real-world deployment but also supports real-time decision-making.

Leveraging the synergy of these technologies can empower organizations to make more informed decisions, implement targeted innovations, and ultimately deliver better care, more efficiently, and at a lower cost. In this context, simulation can act as a bridge between the descriptive and predictive capabilities of these technologies, contributing to the development and implementation of proactive analytics-driven and self-sustainable LHSs.^58,59^

## Take-aways and challenges to leverage simulation in LHSs

Despite the increasing complexity of healthcare organizations and the world in which they operate, the relentless time pressure for improving efficiency and patient outcomes, the massive amounts of data information systems generate, and the added and cheaper computer power, the use of system-wide simulation models in the context of LHSs is just beginning. Among the reasons for the lack of uptake are challenges related to the following.

### Data management

Effective data management within LHSs faces obstacles related to data availability, access, linking diverse sources, reliability, cleaning, and maintenance.^60,61^ While integrated data platforms are a starting point for addressing these issues, significant effort is needed to analyze and standardize data, especially across multiple organizations, to enable meaningful improvements and system-wide learning.

### Digital infrastructure

The digital infrastructure of a LHS should provide essential services that can be applied across various learning cycles.^13^ In regard to simulation, these services should include sophisticated simulation software. Indeed, such applications are needed to support complex analysis and provide the full range of answers that may be needed by decision-makers.^36^ Integrating dedicated software solutions for simulation modelling within a LHS infrastructure would thus be needed to fully leverage its optimization and innovation capabilities.

### Expertise and culture

Successfully leveraging simulation in LHSs requires the right expertise and culture, including knowledge of best practices for selecting appropriate modelling approaches and suitable software. Furthermore, transforming raw healthcare data into useful datasets demands a blend of data science and clinical expertise to ensure the data preparation is both technically sound and clinically meaningful.^61^ Additionally, a broader set of complementary knowledge and roles may be needed throughout the simulation process to ensure its success, from model development to ongoing model governance, including model validation and deployment.^62^ In particular, in-depth practical expertise is needed to integrate simulation outcomes into routine operations. Successful examples of the latter highlight the importance of not only involving senior leadership overall but also of having local leaders and stakeholders that are highly knowledgeable about the system being intervened, motivated to address the issue being studied, and empowered to facilitate the process and implement desired changes.^63,64^

### Costs and benefits

Simulation brings with it costs related to: the use of specialized simulation software; staff time required to develop models, including time spent in data collection and data analysis activities; stakeholder engagement, for example, to support data collection and model validation; and the implementation of the changes suggested by the model.^65^ The benefits of simulation vary based on the specific context and how it is applied. Operational benefits are typically measured in terms of savings (e.g., reduced overtime through better planning) or increased throughput (e.g., ability to serve more patients with the same number of staff and other clinical resources). Health benefits may be measured based on clinical outcomes (e.g., higher number of patients identified in an earlier stage of a disease) or in terms of downstream improvements (e.g., using quality-adjusted life years). While only a few studies report on the return on investment of real-life implementations in healthcare, those that do show high benefit-to-cost ratios.^63,65^

Simulation has the capability to support better-informed, lower-risk decision-making and innovation in LHSs. While fully leveraging simulation requires resources and efforts, the benefits of using simulation techniques promise to outweigh required investments. Moreover, the rapid development of LHSs in Canada creates a timely momentum for integrating simulation as a core method for enabling the transformation of our increasingly digital Canadian health systems and healthcare organizations.
